# Association between peripheral muscle strength, exercise performance, and physical activity in daily life in patients with Chronic Obstructive Pulmonary Disease

**DOI:** 10.1186/2049-6958-9-37

**Published:** 2014-07-03

**Authors:** Anne-Kathrin Rausch-Osthoff, Malcolm Kohler, Noriane A Sievi, Christian F Clarenbach, Arnoldus JR van Gestel

**Affiliations:** 1 Pulmonary Division, University Hospital of Zurich, Zurich, Switzerland; 2 Department of Physiotherapy, Zurich University of Applied Sciences, Winterthur, Switzerland; 3 Centre for Integrative Human Physiology, University of Zurich, Zurich, Switzerland; 4 Zurich University of Applied Sciences, School of Health Professions, Department of Physiotherapy, Technikumstrasse 71, 8401 Winterthur, Switzerland

**Keywords:** Accelerometer, Handgrip-strength, Quadriceps strength, Six-minute-walk-distance, Sit-to-stand-test

## Abstract

**Background:**

Resistance training of peripheral muscles has been recommended in order to increase muscle strength in patients with Chronic Obstructive Pulmonary Disease (COPD). However, whether peripheral muscle strength is associated with exercise performance (EP) and physical activity in daily life (PADL) in these patients needs to be investigated.

The aim of this study is to evaluate whether strength of the quadriceps muscle (QS) is associated with EP and daily PADL in patients with COPD.

**Methods:**

We studied patients with COPD (GOLD A-D) and measured maximal isometric strength of the left QS. PADL was measured for 7 days with a SenseWear-Pro® accelerometer. EP was quantified by the 6-minute walk distance (6MWD), the number of stands in the Sit-to-Stand Test (STST), and the handgrip-strength. Univariate and multivariate analyses were used to examine possible associations between QS, PADL and EP.

**Results:**

In 27 patients with COPD with a mean (SD) FEV_1_ of 37.6 (17.6)% predicted, QS was associated with 6MWD, STST, and handgrip-strength but not with PADL. Multiple linear regression analyses showed that QS was independently associated with the 6MWD (β = 0.42, 95% CI 0.09 to 0.84, p = 0.019), STST (β = 0.50, 95% CI 0.11 to 0.86, p = 0.014) and with handgrip-strength (β = 0.45, 95% CI 0.05 to 0.84, p = 0.038).

**Conclusions:**

Peripheral muscle strength may be associated with exercise performance but not with physical activity in daily life. This may be due to the fact that EP tests evaluate patients’ true abilities while PADL accelerometers may not.

## Background

Physical inactivity in daily life is a prominent feature in patients with Chronic Obstructive Pulmonary Disease (COPD) [1-4] and the amount of physical activity in daily life (PADL) gradually declines with the severity of disease [4-6]. Several studies have demonstrated that the level of PADL is associated with the number of hospitalizations [7-9] and is known as the strongest predictor of all-cause mortality [7,10] in patients with COPD. As a result, both the American Thoracic Society and the European Respiratory Society [11] stressed the fact that long-term self-management and adherence to exercise at home should be the primary goals of pulmonary rehabilitation programs (PR).

One of the extrapulmonary manifestations of COPD is skeletal muscle dysfunction [12-15] and therefore resistance training of peripheral muscles has been strongly recommended during pulmonary rehabilitation (PR) [16,17]. As there is compelling evidence highlighting the role of muscular strength in exercise performance (EP) in patients with COPD [13,18-22], it seems reasonable to assume that enhanced peripheral muscle strength subsequently leads to improvements in performing daily tasks and participating in daily life activities. However, it is currently unclear whether peripheral muscle strength is associated with daily physical activity levels (PAL) in patients with COPD. The methods used to assess PADL in previous studies were very different [8,23-30]. Only very few data are available from studies assessing PADL objectively with a multi-dimensional accelerometer. Furthermore, the results of these studies were conflicting: five studies [8,23-26] demonstrated a positive association between quadriceps strength (QS) and PADL whereas four studies [27-30] did not.

Investigating the role of peripheral muscle strength and its contribution to exercise tolerance may be important for understanding the pathophysiology of exercise limitation and reduced PADL in patients with COPD and may help to define the best therapeutic approach in these patients. Therefore, the purpose of the present study was to examine the relationship between QS and EP (reflected by the 6-minute walk distance (6MWD), the number of stands in the Sit-to-Stand Test (STST), handgrip-strength) and PADL as assessed by a multi-dimensional accelerometer (the SenseWear Pro® armband) in patients with COPD.

## Methods

### Study design

A cross-sectional study in patients with COPD (GOLD-classification A-D) was performed.

Patients with COPD referred to the Pulmonary Division, University Hospital of Zurich, Switzerland between March and September 2012 were considered for participation in the study. The inclusion criteria for patients were: male and female subjects aged 40–75 years and confirmed COPD according to GOLD-guidelines [31]. The participants were included in a convenience sample. The study was approved by the Research Ethics Committee of the University Hospital of Zurich, Switzerland (KEK-ZH-NR: 2011-0106/1) and written informed consent was obtained from all patients.

### Sample size calculation

The sample size calculation was based on the results reported by Mador and colleagues [24].

We calculated that the minimum sample size needed to detect a significant association between QS and PADL with 80% power, a significance level of .05 and a hypothetical drop-out-rate due to technical problems of 25%, would be 23 subjects.

### Measurements

#### Pulmonary function

Spirometry, whole-body plethysmography and diffusion capacity measurements were performed according to the American Thoracic Society (ATS) and the European Respiratory Society (ERS) guidelines with a commercially available system [32,33].

#### Six-minute-walk-test (6MWT)

Patients performed the 6MWT following pulmonary function testing. The 6MWT was conducted in accordance with the ATS guidelines [34]. None of the patients used a walking aid in daily life or during the test. The 6MWT was performed on a 30-meter indoor track by an experienced investigator using standardized encouragement strategy [35]. Subjects were allowed to rest if needed. Measurements of SpO2 were performed using a finger pulse oximeter (TuffSat™, DatexOhmeda, USA, and PureLight® 8000, Nonin Medical Inc., Sweden). SpO2, dyspnoea and leg fatigue [36] were assessed after a resting period of 5 minutes immediately after the test.

#### Sit-to-stand-test

The STST was performed in a distraction-protected environment. A standard chair (46 cm height) without arm supports was used. Subjects were asked to stand up from and sitting down on the chair with arms stationary on the hips, repeating the procedure as many times as possible within 1 minute at a patient-defined pace. The patients had to stand in full extension (knee and hip extension) and sitting in a position with their knees at 90° flexion. The number of completed repetitions was counted [37].

#### Handgrip-strength

Handgrip-strength of the dominant hand was measured with a dynamometer (Hand-Dynamometer Bremshey; Accell Fitness, Almere, Netherlands) as described elsewhere [38].

#### Quadriceps strength

Isometric strength of the left quadriceps muscle group was measured during maximum voluntary contraction (QMVC) with the hips and knees in 90° flexion. QMVC was defined as the highest mean strength that could be sustained over 3 s and expressed in Newton-meters (Nm).

Strength was measured with a strap looped around the left leg just proximal to the ankle and connected to a strain gauge. The strain gauge was connected to the Interface series SM S-Type Load Cell (U.S. & Metric) and the Nexus-10™ device (TMS International BV, Netherlands). All subjects were studied while seated in an adjustable straight-backed chair. Three maximum voluntary contractions were recorded for each patient. The average of the three scores was used for further analysis. After each test the patients were able to rest for a period of two minutes.

#### Physical activity in daily life

Daily Physical Activity was measured by a multisensor accelerometer (SenseWear Pro® armband; BodyMedia, Inc., Pittsburgh, PADL, USA), which was worn on the upper right arm. The device estimates energy expenditure (EE) using measurements from a biaxial accelerometer and sensors that quantify galvanic skin response, heat flux and skin temperature. The biaxial accelerometer records the number of steps per day and the duration of PADL [18]. The number of steps per day, metabolic equivalent (MET), total energy expenditure (TEE), and PAL were used in the present study. PAL was calculated using TEE and sleep expenditure as a surrogate for resting energy expenditure (REE) (PAL = TEE/REE). The patients were instructed to wear the accelerometer continuously during 7 consecutive days, except when bathing or showering. The SenseWear Pro® armband was validated to accurately measure PADL and quantify EE in patients with COPD [39-41].

### Statistics

A statistical software package was used for all calculations (SPSS® for Windows, Version 20.0, SPSS Inc., Chicago, IL, USA). Descriptive data for continuous variables are expressed as mean, standard deviation and percentages for frequencies. Variables were tested for parametric distribution by applying the Shapiro-Wilk Test (null hypothesis rejection set at p < 0.25). Univariate regression was performed to evaluate a possible association between QS and measures reflecting PADL derived from accelerometry, 6MWD, the number of stands during the STST, and handgrip-strength. The multivariate analysis was performed to analyse if QS is independently associated with these measures reflecting PADL and EP. The multivariate analysis included measures of PADL that showed significant associations in the univariate regression analysis, Forced Expiratory Volume in 1 second (FEV_1_) and age. A p < 0.05 was considered to indicate statistical significance.

## Results

**Figure 1:**
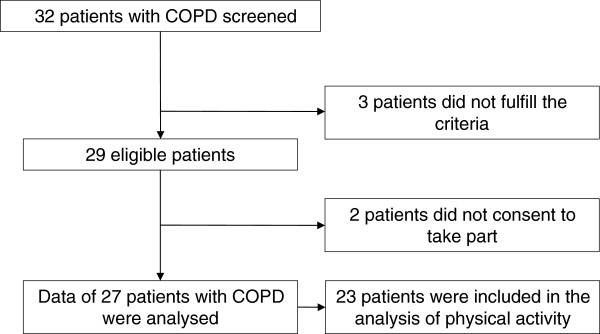
Study profile.

Figure 1 shows the study profile. Twenty-seven patients (thirteen females) with COPD and an average age of 62 ± 5 years agreed to take part and were included in the study. Anthropometrical characteristics and data reflecting pulmonary function, EP and PADL are presented in Table 1. Patients reported the following comorbidities: diabetes mellitus, osteoporosis, hypertension, depression, arthritis, and peripheral arterial disease. Five of participating subjects declared to be active smokers. The majority of patients (74.1%) were “at high risk and with high symptom burden” (GOLD D).

**Table 1:** Anthropometrical characteristics and pulmonary function, EP and PADL data of the study group

**Variable**	
***Anthropomertrics***	
Subjects (n)	27
Female/male	11/16
Age (years)	62.3 (5.7)
BMI (kg/m2)	24.5 (5.8)
***GOLD-Classification***	
A	3.7%
B	18.5%
C	3.7%
D	74.1%
***Pulmonary function***	
FEV_1_ (l)	1.1 (0.6)
FEV_1_ (% predicted)	37.6 (17.6)
FEV_1_/FVC (ratio)	0.40 (0.14)
TLC (% predicted)	112.6 (25.4)
RV/TLC	56.8 (10.7)
PaO_2_ (kPa)	9.5 (1.9)
PaCO_2_ (kPa)	5.1 (0.5)
***Exercise performance***	
6MWD (m)	390 (103)
**Sit-to-Stand Test (n)**	20 (7)
Handgrip Test (kg)	33.7 (10.3)
Quadriceps strength (Nm)	14.5 (5.2)
***Daily physical activity by accelerometry***	
PAL (ratio)	1.44 (0.16)
MET (kcal/h/kg)	30.3 (4.7)
TEEACC (kcal/day)	2222 (467)
Steps/day (n)	4097 (2325)
Time <3METs (min)	1341 (876)
Time 3–6 METs (min)	86 (30)
Time >6 METs (min)	0 (2)

Values are presented as mean (SD). BMI, body mass index; DL_CO_, diffusion capacity for carbon monoxide; FEV_1_, forced expiratory volume in one second; FEV_1_/FVC ratio, forced expiratory volume in 1 sec (FEV_1_) expressed as percent of FVC; MET, metabolic equivalent; PAL, physical activity level; PaCO_2_, partial pressure of carbon dioxide; PaO_2_, partial pressure of oxygen;RV/TLC, residual volume/total lung capacity ratio; TEEACC, total energy expenditure per day as assessed by accelerometry; TLC, total lung capacity.

### Physical activity

In four of the 27 patients PADL data were unavailable due to technical problems. Therefore, the analysis of PADL data included 23 patients. The mean PAL of the patients was 1.44 (0.16) (Table 1); 41% of the patients had an extremely inactive lifestyle (PAL < 1.4), 55% had a sedentary lifestyle (PAL 1.40-1.69) and 4% were classified as moderate to vigorously active (PAL ≥ 1.70). Mean total energy expenditure (TEEACC) estimated by accelerometry was 2222 (467) kcal/day.

### Relationship between quadriceps strength, exercise performance and physical activity

**Figure 2:**
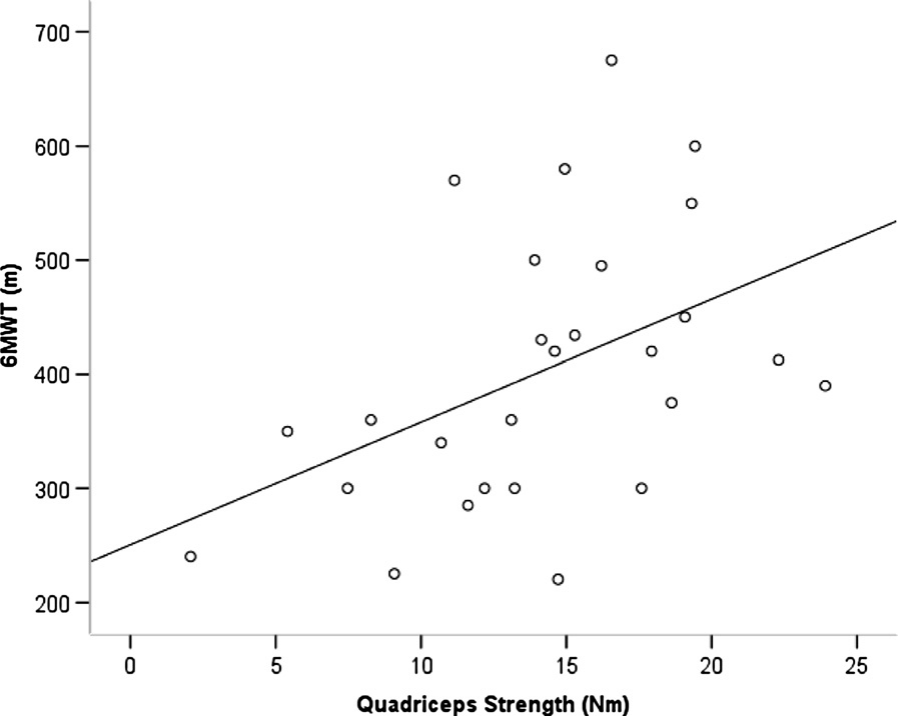
Scatterplot showing the relationship between quadriceps strength and the 6-minute walk distance, 6MWD (β = 0.45, 95% CI 0.085 to 0.835, p = 0.018).

There was a statistically significant positive association between QS and the 6MWD (Figure 2), the number of stands during the STST, and handgrip-strength, but not with PADL as assessed by the SenseWear Pro® armband (Table 2).

**Table 2:** Associations with quadriceps strength

**Variable**	**Coefficient β**	**95% CI**	**p**
***Daily physical activity***			
MET (kcal/24 h/kg)	0.100	-0.371 – 0.582	0.650
TEEACC (kcal/day)	0.274	-0.171 – 0.749	0.206
PAL	0.092	-0.345 – 0.516	0.684
Steps/day (n)	-0.085	-0.567 – 0.387	0.699
***Exercise performance***			
6MWD (m)	0.451	0.085 – 0.835	0.018
Sit-to-Stand test (n)	0.502	0.105 – 0.857	0.015
Handgrip test (kg)	0.455	0.050 – 0.837	0.029
Time < 3METs (min)	-0.055	-0.811 - 0.434	0.822
Time 3–6 METs (min)	0.449	-0.111 – 1.010	0.080

Univariate regression is expressed as β. 6MWD, 6-minute walk distance, MET, metabolic equivalent; PAL, physical activity level; TEEACC, total energy expenditure per day as assessed by accelerometry. P < 5% is significant.

The results of the multivariate analyses are shown in Tables 3, 4 and 5. After correction for age and FEV_1_, QS was found to be independently associated with the number of stands during the STST (β = 0.50, 95% CI 0.11 to 0.86, p = 0.014) and handgrip-strength (β = 0.45, 95% CI 0.05 to 0.84, p = 0.038). Both FEV_1_ and QS were independently associated with the 6MWD (QS: β = 0.42, 95% CI 0.09 to 0.84, p = 0.019). 52.4% of the variance in 6MWD could be explained by the model (Table 3). QS explained 50.2% and 45.5% of the variance in the number of stands during the STST and handgrip-strength, respectively (Tables 4 and 5).

**Table 3:** Multiple regression analysis of predictors of the 6MWD (m)

**Model 1**	**Coefficient B**	**Std. Error**	**Coefficient β**	**t**	**p**
Residual	434.893	162.825		2.67	0.014
Quadriceps muscle strength (Nm)	10.02	3.96	0.42	2.53	0.019
FEV_1_ (% predicted)	2.29	1.04	0.371	2.20	0.038
Age (years)	-4.30	2.56	-0.28	-1.68	0.107

6MWD, 6-minute walk distance; FEV_1_, forced expiratory volume in one second (r2 = 0.524).

**Table 4:** Multiple regression analysis of predictors of the number of stands (STST)

**Model 2**	**Coefficient B**	**Std. Error**	**Coefficient β**	**t**	**p**
Residual	14.71	13.19		1.12	0.279
Quadriceps muscle strength (Nm)	0.85	0.31	0.50	2.71	0.014
FEV_1_ (% predicted)	0.15	0.10	0.28	1.52	0.146
Age (years)	-0.22	0.19	-0.22	-1.19	0.250

FEV_1_, forced expiratory volume in one second (r2 = 0.502), STST, Sit-to-Stand Test.

**Table 5:** Multiple regression analysis of predictors of handgrip-strength

**Model 3**	**Coefficient B**	**Std. Error**	**Coefficient β**	**t**	**p**
Residual	24.13	18.15		1.330	0.199
Quadriceps muscle strength (Nm)	0.96	0.42	0.45	2.23	0.038
FEV_1_ (% predicted)	0.07	0.11	0.13	0.52	0.517
Age (years)	-0.11	0.27	-0.85	-0.42	0.680

FEV_1_, forced expiratory volume in one second (r2 = 0.455).

## Discussion

In 27 patients with COPD (GOLD A-D) QS was independently associated with the 6MWD, the number of stands in the STST, handgrip-strength but not with PADL as assessed by the SenseWear Pro® armband.

It has been recognized that skeletal muscle dysfunction is a common feature in patients with COPD, and may play a significant role in morbidity and mortality [42]. Several authors [19,43,44] have demonstrated that peripheral muscle weakness may be a major contributor to exercise limitation in COPD. Killian and colleagues [22] measured leg effort and dyspnoea in 97 patients with chronic airway obstruction during maximal cycle ergometry. Indeed, patients complained more about leg discomfort than about dyspnoea after cycle ergometry. In accordance with these results, in this study we found that QS was independently associated with exercise performance (EP) as quantified by several laboratory-based tests.

International guidelines recommend exercise training to improve skeletal muscle function as an essential component of PR in patients with COPD [11,16]. However, in order to be meaningful for patients with COPD, improvements in peripheral muscle strength need to be translated into changes in PADL and participation in everyday situations. As there is compelling evidence highlighting the role of muscular strength in the EP in patients with COPD, it seems reasonable to assume that impaired muscle strength subsequently leads to difficulties in performing daily tasks and participating in daily life activities. However, we failed to demonstrate an association between QS and any measure reflecting PADL as assessed by the SenseWear Pro® armband in the patients of our study.

After reviewing the literature, a total of nine studies, which investigated the association between QS and PADL in patients with COPD were found. Out of nine studies only two [25,27] used a multi-sensor accelerometer to objectively quantify PADL. Five studies [8,23-26] succeeded to demonstrate an association between QS and PADL and four [27-30] failed to demonstrate such association.

In this study QS was independently associated with EP but not with PADL as assessed by the SenseWear Pro® armband. Although the design of this study does not allow to establish a causal relationship between QS and PADL, these results need to be elucidated. In a previous research we demonstrated that EP tests do not necessarily reflect daily activities in patients with COPD accurately [45]. This may be due to the fact that EP tests evaluate patients’ true abilities while PADL accelerometers may not. Differences may depend on personal factors, such as effort spent, time for leisure activities and motivation, but also external factors such as the instructions and the encouragement given to the participants during EP testing in a clinical setting [45]. This may explain the fact that QS is not associated with PADL in the patients of our study.

It should be stressed that correlations do not prove causal relationships. Interventional studies investigating the effect of PR- induced improvements in peripheral muscle strength on daily PADL allow for a more precise and meaningful examination of the association between QS and PADL. Although the main purpose of PR should be enhancing PADL [31], only few studies have investigated the impact of PR on PADL in patients with COPD. Pitta and colleagues [46] assessed PADL at baseline, after three months and at the end of a six-month multidisciplinary rehabilitation program in 29 patients with COPD. Although three months of PR improved exercise capacity and QS, these improvements did not result in patients spending more time walking in daily life. After additional three months of PR, PADL did actually increase [46]. However, it would be more powerful to analyse if PR-induced improvements in QS are associated with improvements in PADL in patients with COPD. In the only study that addressed this issue [24], PR-induced improvements in QS did not predict changes in PADL. This is in good accordance with the findings of the present study, that QS is not related to the level of PADL in patients with COPD.

### Limitations

The present study has certain limitations that need to be taken into account. The number of subjects is small given the variance in data. In addition, the majority of the study population represents GOLD D, which may affect outcomes. We did not investigate potential exercise performance limiting factors such as hemodynamic factors, cardiac autonomic nervous system factors, and psychological factors (motivation, anxiety and depression). Neither did we control for the use of long-acting β_2_-agonists, which may have a small effect on exercise performance and PADL. Furthermore, because cardiopulmonary exercise testing (CPET) is regarded as the gold standard assessment for evaluating exercise capacity, the tests used in this study may not reflect the true exercise capacity. It should be stressed that the results of the STST mainly reflect the performance of lower extremity muscles.

## Conclusions

We conclude that quadriceps strength may be associated with exercise capacity as assessed by several laboratory-based tests such as the 6-minutes walking test, the sit-to-stand test and handgrip-strength. Future well-designed interventional trials investigating the predominant role of peripheral muscle strength on daily physical activity are needed to establish the preliminary finding of the current study. However, quadriceps strength was not associated with physical activity levels in patients with COPD. This may be due to the fact that patients with COPD do not exhaust their functional abilities during daily living, as they do during laboratory-based testing.

## Competing interest

None of the authors has a conflict of interest related to the content of the manuscript.
